# Patterns of HIV, TB, and non-communicable disease multi-morbidity in peri-urban South Africa- a cross sectional study

**DOI:** 10.1186/s12879-015-0750-1

**Published:** 2015-01-17

**Authors:** Tolu Oni, Elizabeth Youngblood, Andrew Boulle, Nuala McGrath, Robert J Wilkinson, Naomi S Levitt

**Affiliations:** Division of Public Health Medicine, School of Public Health and Family Medicine, University of Cape Town, Room 2.24, Entrance 5, Falmouth building Anzio road, Observatory 7925, Cape Town, South Africa; Health Impact Assessment Directorate, Western Cape Department of Health, Cape Town, South Africa; Centre for Infectious Disease Epidemiology Research, Division of Public Health Medicine, School of Public Health and Family Medicine, University of Cape Town, Cape Town, South Africa; Department of Medicine, University of Cape Town, Cape Town, South Africa; Academic Unit of Primary Care and Population Sciences, University of Southampton, Southampton, UK; Africa Centre for Health and Population Studies, University of Kwazulu Natal, Durban, South Africa; Department of Medicine, Imperial College, London, W2 1PG UK; Medical Research Council National Institute of Medical Research, London, NW7 1AA UK; Chronic Disease Initiative for Africa, Cape Town, South Africa

**Keywords:** HIV, Tuberculosis, Hypertension, Diabetes, Multimorbidity

## Abstract

**Background:**

Many low and middle-income countries are experiencing colliding epidemics of chronic infectious (ID) and non-communicable diseases (NCD). As a result, the prevalence of multiple morbidities (MM) is rising.

**Methods:**

We conducted a study to describe the epidemiology of MM in a primary care clinic in Khayelitsha. Adults with at least one of HIV, tuberculosis (TB), diabetes (DM), and hypertension (HPT) were identified between Sept 2012-May 2013 on electronic databases. Using unique patient identifiers, drugs prescribed across all facilities in the province were linked to each patient and each drug class assigned a condition.

**Results:**

These 4 diseases accounted for 45% of all prescription visits. Among 14364 chronic disease patients, HPT was the most common morbidity (65%). 22.6% of patients had MM, with an increasing prevalence with age; and a high prevalence among younger antiretroviral therapy (ART) patients (26% and 30% in 18-35 yr and 36–45 year age groups respectively). Among these younger ART patients with MM, HPT and DM prevalence was higher than in those not on ART.

**Conclusions:**

We highlight the co-existence of multiple ID and NCD. This presents both challenges (increasing complexity and the impact on health services, providers and patients), and opportunities for chronic diseases screening in a population linked to care. It also necessitates re-thinking of models of health care delivery and requires policy interventions to integrate and coordinate management of co-morbid chronic diseases.

## Background

The concept of multi-morbidity (MM), defined as the co-existence of more than one chronic condition in one person, is well recognized, usually within the context of older age [1]. Patients with MM have increased utilization of health care, a reduced quality of life and poorer health outcomes [1-4]. A recent systematic review of MM patterns described a non-random pattern of MM for which common pathophysiological mechanisms underlie each disease constellation [1]. However all studies in this review were conducted in high-income settings, predominantly in older age populations, and included only non-communicable diseases (NCD). In low- and middle-income countries (LMIC), with burgeoning urbanisation, not only is the prevalence of NCD increasing, it is occurring alongside chronic infectious diseases. Thus patterns of MM will differ.

South Africa is the most urbanised country in sub-Saharan Africa with 62% of the country’s population living in cities [1, 5]. The rapid and unplanned nature of this demographic shift affects life choices and opportunities; contributing to epidemiological transition with an increase in unhealthy dietary patterns, a decrease in physical activity and a rising NCD burden [2-4, 6, 7]. South Africa has the highest burden of hypertension (HPT) in the >50 years old population and among the highest type 2 diabetes mellitus prevalence in sub-Saharan Africa [1, 8, 9]; this is predicted to increase further over the next few decades. Against this background, the burden of HIV and tuberculosis (TB) remain high. Effective antiretroviral therapy (ART), in widespread use in South Africa since 2005/6 has resulted in increasing survival and ageing among HIV-infected persons and an accompanying rise in NCD co-morbidities in this sub-group [10, 11]. Furthermore, the premature ageing effect of HIV will likely further contribute to multiple morbidities in the population [12], at younger ages than described in low HIV-burden settings. The morbidity and mortality rates for NCD, HIV and TB in South Africa disproportionately affects poor people with the NCD burden fuelled by a high prevalence of obesity, which affects 40% of the adult female population [7]. However, little is known about the prevalence and patterns of MM in South Africa and other LMIC, where the prevalence of NCD is rising alongside established HIV/TB epidemics.

A better understanding of the patterns of chronic infectious and non-communicable disease multimorbidity (MM) in LMIC is therefore required to develop strategies to prevent and better manage these co-existing and interacting conditions. A study conducted in the UK comparing measures of MM found the number of prescribed drugs to be the most powerful measure for predicting future healthcare utilization and second most powerful for predicting mortality [13]. We aimed to use routine data from a public health programme to explore the distribution of chronic diseases and patterns of HIV, TB, and NCD MM in adults who have received care and treatment in a public clinic.

## Methods

Setting: We conducted a cross-sectional study in Michael Mapongwana clinic, a primary care health facility in Khayelitsha, an informal township near Cape Town with a population of >500 000 predominantly black Africans. This study was approved by the University of Cape Town, Faculty of Health Sciences Human Research Ethics committee (HREC Ref no: 493/2014).

Data Source: Data on treatment prescriptions were extracted from two routine electronic databases. Patients who are considered stable on chronic disease medication receive their monthly prescriptions through the Chronic Disease Dispensing unit (CDU), an outsourced centralised unit that collects prescriptions for stable chronic patients from health facilities, dispenses the medicines, and returns them to the facilities which the patients attend, packaged in tamper-proof parcels. A record of medicines dispensed is kept on a database that is sent to the Western Cape Department of Health Data Repository on a monthly basis. The second database used is the electronic prescription system that manages pharmacy prescriptions electronically. This system has been in use across secondary and tertiary-level hospitals in the Western Cape province for >10 years and enables pharmacy records linked to an individual patient to be accessible across hospitals. Roll out of this system in primary care clinics began in September 2012 in Michael Mapongwana clinic in Khayelitsha. This database captures chronic disease patients not receiving medicines through the CDU, including sub-optimally managed chronic disease patients. Every patient accessing health care in the public clinics and hospitals is ascribed a unique patient master index (PMI) that serves to longitudinally link prescriptions across different databases and health care facilities.

Persons prescribed medicines for at least one of the four most prevalent chronic diseases (HIV, TB, diabetes (DM), HPT) were identified from the electronic pharmacy and CDU databases from September 2012, when the electronic pharmacy database was launched, to May 2013. This time period was selected to capture 6-monthly prescriptions over a 9-month period from the electronic pharmacy databases. The anonymised dataset extracted included age, sex, and medications prescribed at all consultations over the study period. Using the PMI, medicines prescribed across all health facilities in the province were linked to each patient. Each drug class was assigned a condition based on South African prescription guidelines: HPT defined as a prescription of at least one of hydrochlorthiazide, enalapril, or amlodipine; DM defined as a prescription of metformin, gliclazide, glibenclamide, or insulin; HIV/ART defined as prescription of ART; TB defined as prescription of rifampicin, isoniazid, or pyrazinamide. MM was defined as receiving medication for two or more of the 4 morbidities measured.

Statistical analysis: Descriptive analyses were represented using percentages, frequencies, and tabulation. Age categories 18–35, 35–45, 46–55, >55 were used to explore the age-distribution of chronic diseases and MM; stratified by sex using the λ^2^ test. The prevalence of MM was also calculated stratified by HIV/ART status, into ART and non-ART (HIV status unknown) groups, across the different age groups. Co-morbidity patterns across the individual chronic diseases were examined. The λ^2^ test was used to measure differences in the prevalence of chronic diseases and MM. The Shapiro-Wilk test was used to test for normality and the Kruskal Wallis test used to test for statistical significance of non-parametric continuous variables. Significance testing was done using 2-sided p-values and 95% confidence intervals. All data were analysed using STATA 12.0 (StataCorp, College Station, TX, USA).

Role of funding sources: The funders had no role in the design, collection, analysis, and writing of this manuscript. TO confirms that she had full access to all data and had final responsibility for the decision to submit the manuscript for publication.

## Results

### A. Baseline characteristics and descriptive analysis

**Figure 1:**
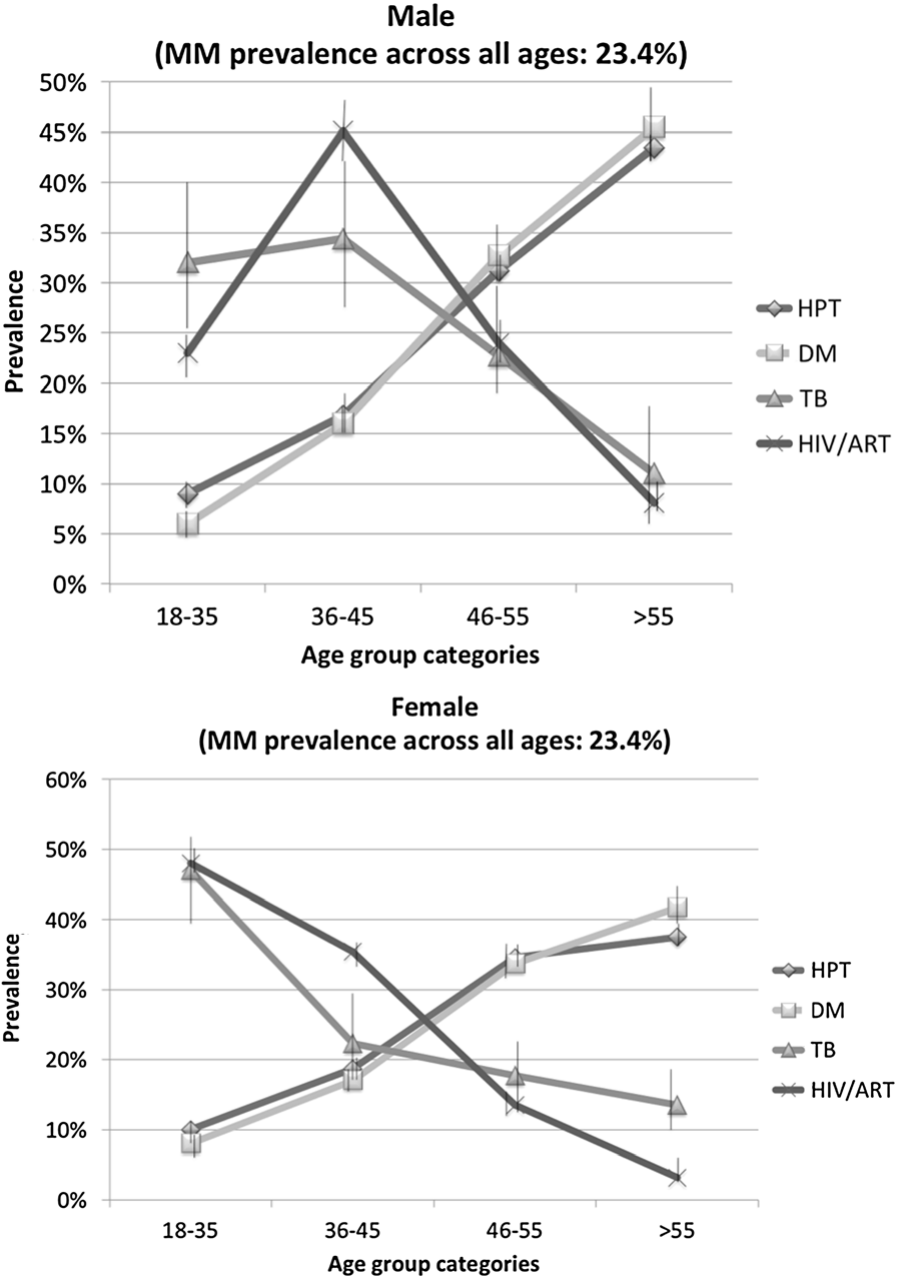
**Distribution of HPT, DM, HIV/ART, and TB, stratified by gender across age groups among patients with prescriptions for at least one of HPT, DM, HIV/ART, and TB.** Key: HPT Hypertension; DM Diabetes; ART Antiretroviral therapy; TB Tuberculosis; MM Multimorbidity.

A total of 32 474 patients attended the clinic and received at least one prescription between Sept 2012 and May 2013. Of these, 14 700 (45%) were consultations for HIV, TB, DM or HPT. Three hundred and thirty six of the 14 700 patients were aged <18 years and excluded from further analysis. The final study population sample size was 14 364 adults. The age distribution of the study population showed that while 71% of the total population were female, the male: female ratio increased with increasing age (Table 1) with a higher proportion of male: female ratio in the 46–55 and >55 years age groups. The median age was 46 years (interquartile range (IQR) 36–56) overall; 46 years (IQR 35–55) in females and 48 years (IQR 39–57) in males. The prevalence of HPT, DM, HIV and TB in the study population, overall and stratified by gender is summarized in Table 1. As shown in Figure 1 there was an increase in the prevalence of NCD (HPT and DM) with increasing age. Overall, the prevalence of HPT, DM and TB was higher in male versus female participants. However, analysed by gender, it was evident that younger females had a higher prevalence of DM (18–35 age group; 7.5% vs. 5.8%, p = 0.015) and HPT (26–35 (9.2% vs. 8.5%, p < 0.0001), 36–45 (18.7% vs. 16.8%, p < 0.0001), and 46–55 (34.6% vs. 31.2%, p < 0.0001) age groups). Men >55 had a higher prevalence of HPT (43.5% vs. 37.5%, p < 0.001) and HIV/ART (8.1% vs. 3.2%, p < 0.0001).

**Table 1:** **Baseline characteristics, overall and stratified by gender**

		**Female%**	**Male%**	**Total%**	
		**N = 10231**	**N = 4119**	N = 14350*	
		**(95% C.I.)**	**(95% C.I.)**	**(95% C.I.)**	
**Age**	18-35	24.4 (23.5-25.2)	14.0 (12.9-15.1)	21.4 (20.7-22.1)	
	36-45	24.8 (23.9-25.6)	26.6 (25.3-28)	25.3 (24.6-26.0)	
	46-55	26.1 (25.3-27.0)	28.4 (27.1-29.8)	26.8 (26.1-27.5)	
	>55	24.7 (24.0-25.7)	31.0 (29.6-32.4)	26.6 (25.9-27.3)	
	Total	N = 10231	N = 4119		
**Median age**	Years (IQR)	46 (35–55)	48 (39–57)	46 (36–56)	
**Hypertension**		63.9 (63.0-64.9)	66.3 (64.8-67.7)	64.6 (63.8-65.4)	
**Diabetes**		17.7 (17.0-18.5)	19.6 (18.4-20.8)	18.3 (17.6-18.9)	
**HIV/ART**		39.5 (38.5-40.4)	35.7 (34.3-37.2)	38.4 (37.6-39.2)	
**Tuberculosis**		2.3 (2.1-2.6)	3.7 (3.2-4.4)	2.7 (2.5-3.0)	

*Gender assignment missing for 14 patients.

The distribution of TB and HIV/ART prevalence also differed by gender. Females in the 18–35 age group had a higher HIV/ART (48% vs. 22.8%, p < 0.0001), and TB (46.6% vs. 31.8%, p < 0.0001) prevalence compared to men. By contrast, male patients aged 36–45 and 46–55 had a higher prevalence of HIV/ART (45.1% vs. 35.4%, p = 0.021; and 24% vs. 13.5%, p < 0.0001, respectively) and TB (34.4% vs. 22.3%, p < 0.0001; and 22.7% vs. 17.7%, p = 0.004, respectively) compared to females (Figure 1).

### B. Burden and distribution of multimorbidity

**Figure 2:**
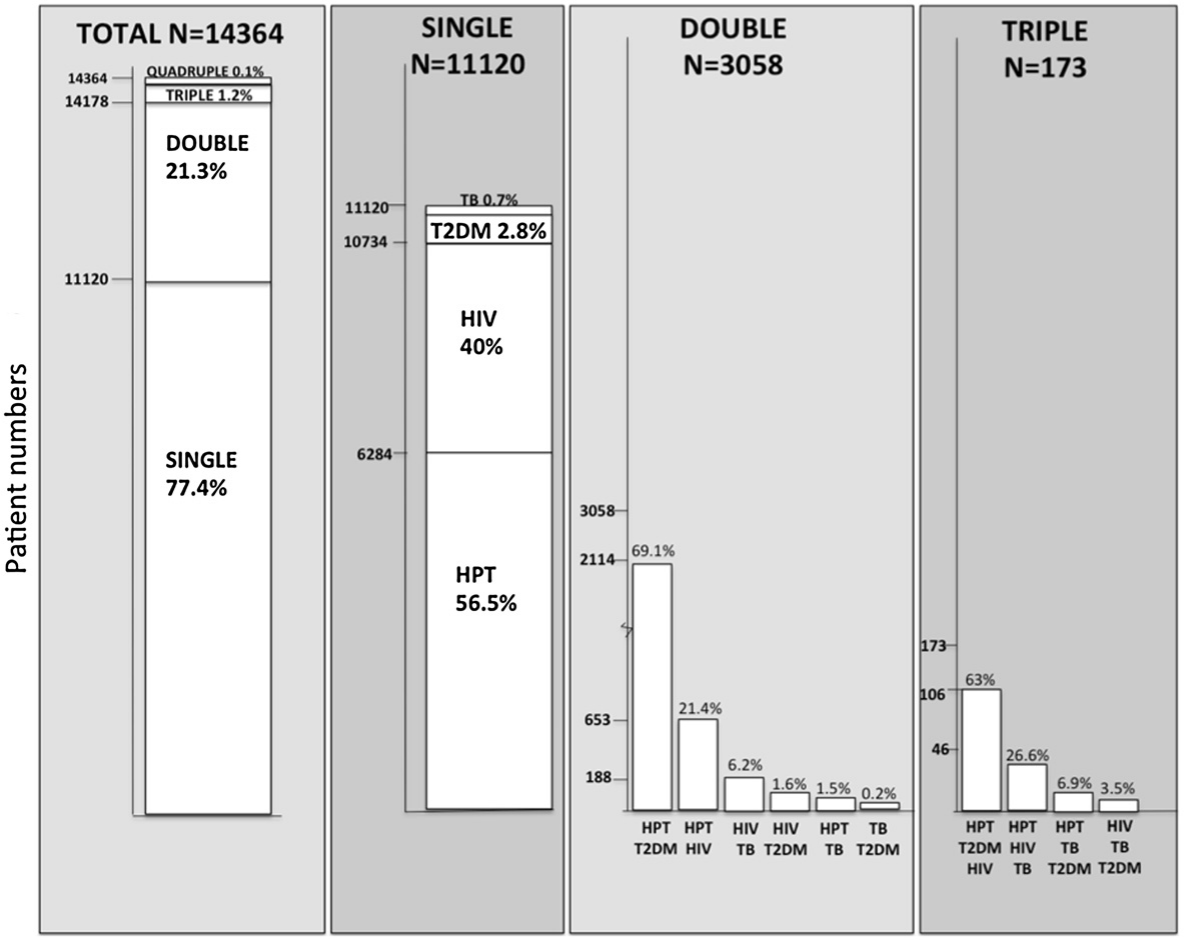
**Patterns and distribution of single, double and triple morbidities.**

**Figure 3:**
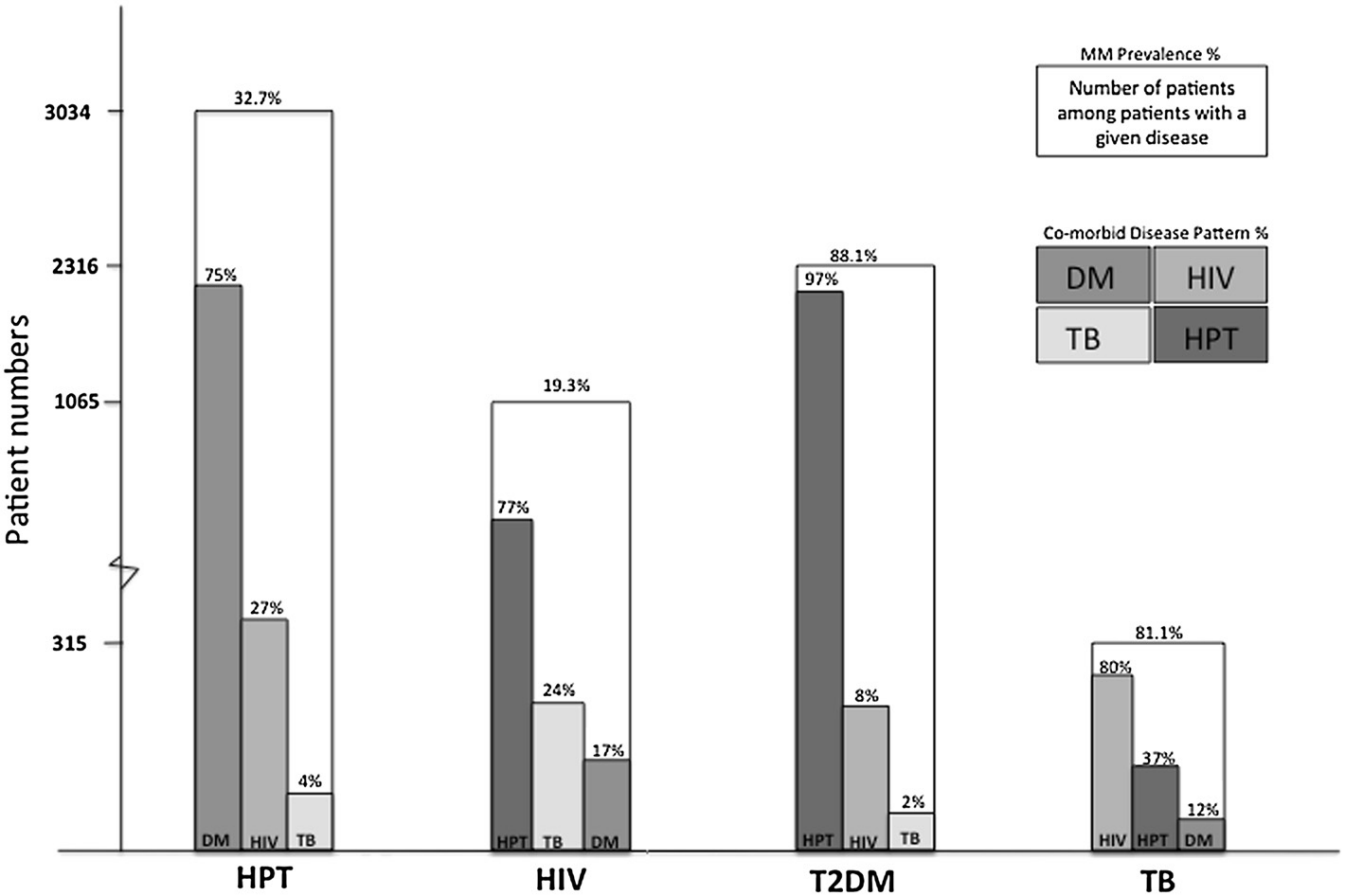
**Proportion of patients with multimorbidity among 32 474 patients who attended the clinic and received any prescription; and the distribution of morbidities among patients with prescriptions for at least one of HPT, DM, HIV/ART, and TB.** Key: HPT Hypertension; DM Diabetes; HIV/ART HIV infected patients on antiretroviral therapy; TB Tuberculosis; MM Multimorbidity.

The overall prevalence of MM was 22.6% (n = 3246) with no significant difference between sexes. The prevalence of MM increased with increasing age. Among patients with MM, 94% had 2 morbidities, the most common combination of which was HPT and DM (Figure 2). Five percent had 3 conditions of which HPT, DM, and HIV were the most common. There was no significant difference in the proportion of MM patients with double, triple, or quadruple morbidities between sexes (data not shown). The overall age distributions were similar, with the highest prevalence in the 36–45 and 46–55 age groups, across these MM categories (data not shown). Although the prevalence of MM was highest in DM patients (88.1%), the number of patients with MM is highest in HPT patients (32.3% of 9 279 patients) due to the high overall prevalence of HPT (65%) of the sample population. The co-morbidity pattern differed across the 4 diseases (Figure 3). Among DM MM patients, 97% had HPT as a co-morbidity, while 75% of HPT MM patients were on DM treatment. Among TB MM patients (81.1% of all TB patients), HIV/ART was the most common co-morbidity (followed by HPT and DM); while HIV/ART MM patients were most commonly receiving HPT treatment (Figure 3).

### C. Age-specific multimorbidity and the effect of HIV

**Figure 4:**
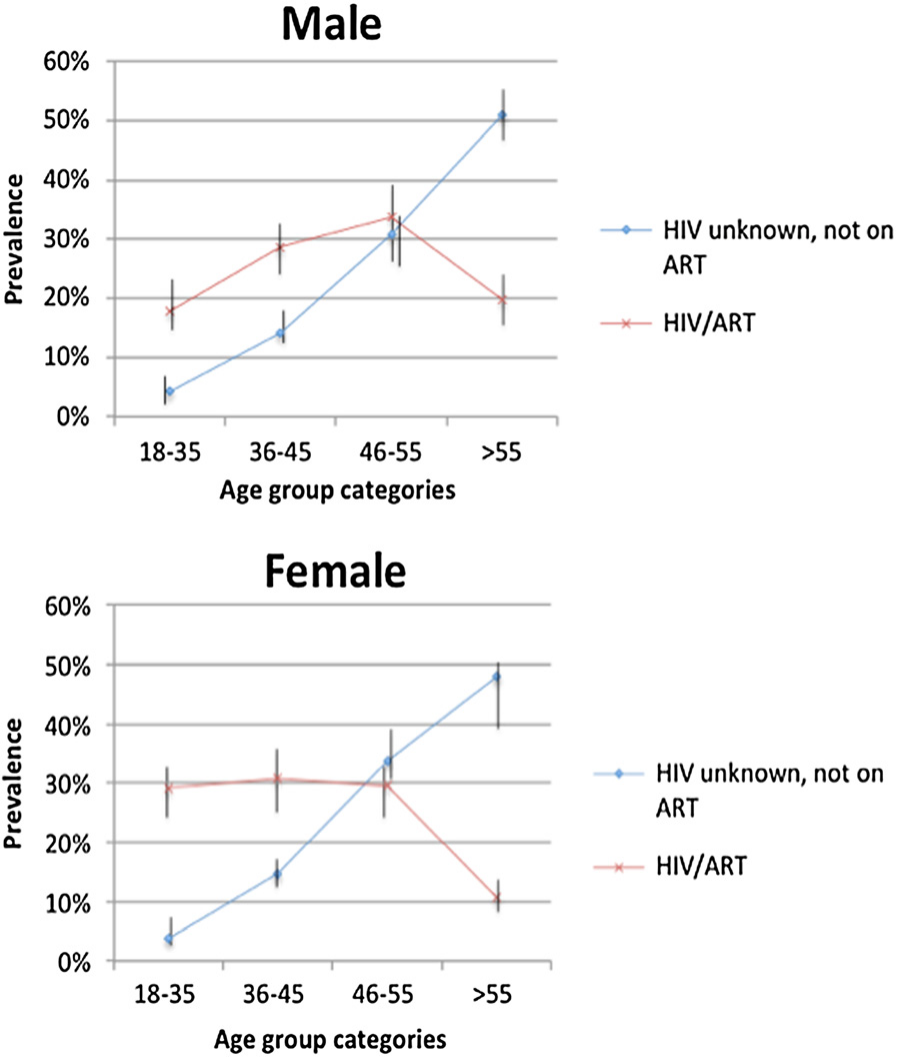
**Distribution of non-HIV morbidities among MM patients (n = 3246), stratified by sex and HIV/ART groups.** Error bars show 95% confidence intervals.

**Figure 5:**
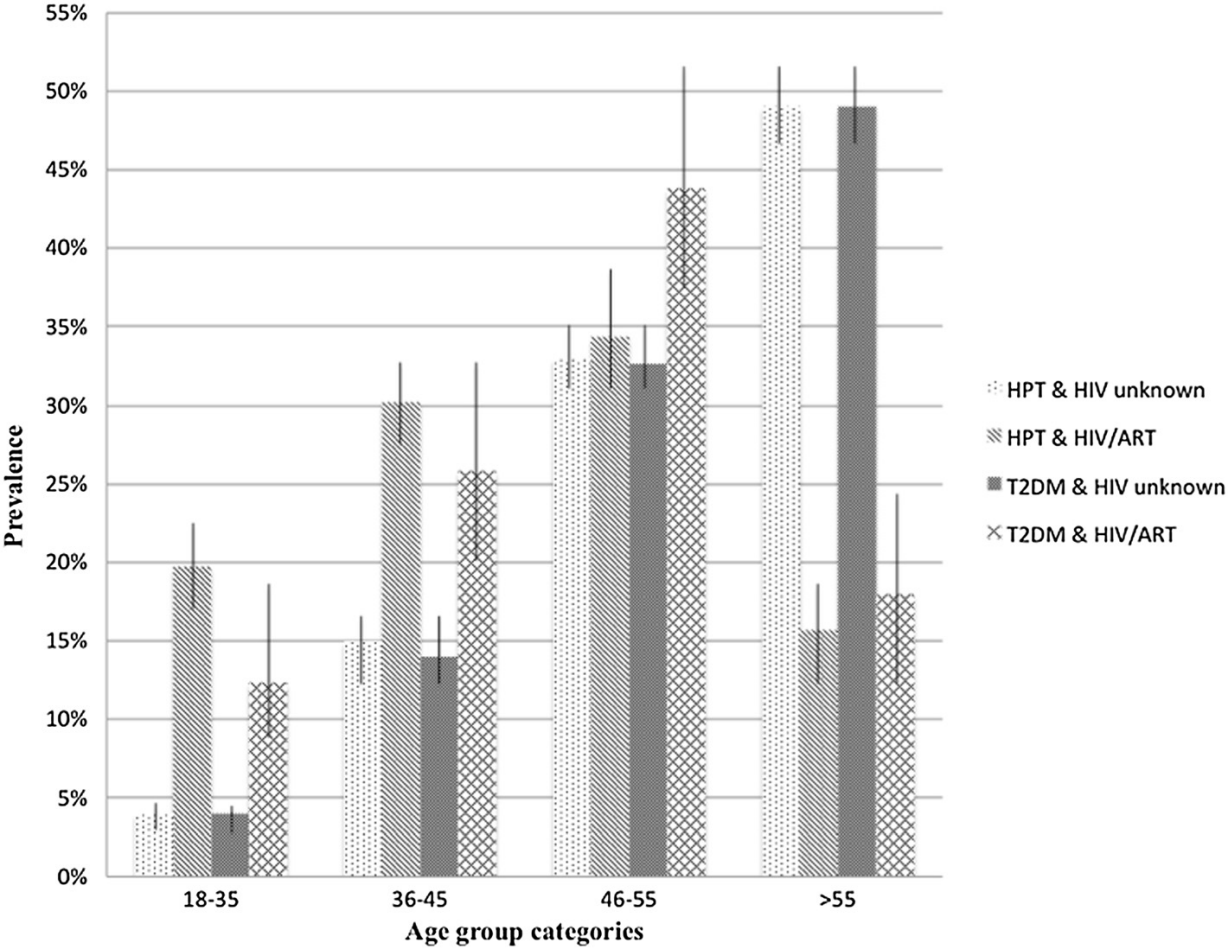
**Distribution of HPT and DM across age groups comparing HIV/ART MM patients to MM patients not on ART (HIV status unknown).** Error bars show 95% confidence intervals.

The prevalence of MM among younger HIV-infected patients in the 18–35 and 36–45 age groups was higher than in their counterparts not on ART, but not in the >55 age group, where the pattern was reversed; although the prevalence of HIV/ART in this age-group was low (Figure 4). Stratified by gender, there was a difference in the age distribution of HIV/ART MM patients with a higher MM prevalence in the youngest the 18–35 age group in females versus male patients (Figure 4; p = 0.017). By contrast, the peak in MM for male ART patients occurred in the older 46–55 age group. Further investigation of the pattern of co-morbid conditions in HIV/ART patients with MM versus MM patients not on ART (HIV status unknown) in the 18–35 years age category revealed a higher prevalence of HPT (19.7% (95% confidence interval (C.I.) 3.1-4.7%) vs. 3.8% (95% C.I. 17.2-22.6%)) and DM (12.3% (95% C.I. 8.3-18.0%) vs. 3.8% (95% C.I. 3.0-4.6%)) in ART MM patients. A similar pattern for HPT (30.2% (95% C.I. 27.2-33.45%) vs. 14.6% (95% C.I. 13.1-16.1%)) and DM (25.8% (95% C.I. 20.0-32.7%) vs. 14.3% (95% C.I. 12.9-15.9%)) was found among MM patients in the 36–45 years age category (Figure 5). In the next age group, 46–55 years, DM co-morbidity remained higher in HIV-infected persons on ART than the non ART group (43.8% (95% C.I. 36.7-51.2%) versus 32.8% (95% C.I. 30.9-34.8%)) but the prevalence of HPT co-morbidity did not differ between the two groups.

## Discussion

This study examined co-morbidity patterns in a cohort of patients receiving at least one prescription for one of 4 diseases over a 9-month period, and has a number of major findings. Firstly, a quartet of chronic diseases accounted for 45% of all consultations in a community health centre providing primary care services for people of low socioeconomic status in Cape Town. Secondly, we found a 22.6% prevalence of MM among chronic disease patients and an associative pattern of MM, with HPT and DM often co-existing. Furthermore HPT was the most common co-morbidity in both HIV/ART and DM patients. Thirdly, we demonstrated that while HIV/ART was the most common co-morbidity among TB patients, 37% and 12% of multi-morbid TB patients were also on treatment for HPT and DM respectively. Fourthly, we found a high prevalence of MM in younger patients on ART (26% and 30% in 18–35 year and 36–45 year age groups respectively). Lastly, we showed that among these younger HIV/ART patients with MM, HPT and DM prevalence was significantly higher than patients in the same age groups who were not on ART.

Our results reveal a very high prevalence of HPT among chronic disease patients. This is congruent with national data that shows a high overall prevalence of HPT in the general population and that HPT is the commonest reason for attendance of primary health clinics in South Africa [14]. A national, population-based study of persons 50 years and older in South Africa reported HPT prevalence of 77.3% [15]. It is noteworthy that HPT was the most common co-morbidity in both DM and HIV/ART MM patients. It was not surprising that 88% of all DM MM patients were also on treatment for HPT; DM patients are routinely screened for HPT in this setting. On the other hand, blood pressure screening is not currently routine in the management of HIV/ART and TB patients. Further the finding that 75% of HPT patients with MM were on treatment for DM suggesting that HPT patients should also routinely be screened for DM, which is not current local practice. Our results are comparable to data from high-income settings. A recent study from Ireland examined co-morbidity patterns (including hypertension, heart disease, arthritis, depression, and chronic lung disease) and reported that 90% of a cohort of DM patients had another morbidity and 66% had HPT [16]. However, 90% of study participants in this study were >50 years old and HIV status was not documented.

Among TB patients with other chronic disease co-morbidities, the prevalence of HIV co-infection was high. This was an expected result as the HIV/TB co-infection rate in this local setting has previously been estimated as 67% [17]. Our data demonstrated that in addition to HIV, TB patients also have a significant prevalence of HPT and DM. A study in Brazil examined chronic disease multimorbidity in TB patients. Whilst they reported a lower prevalence of MM than we found (1.14%; higher in older age groups), their results highlighted the importance of MM as they demonstrated that death from causes other than TB was higher, and cure rates lower in TB-MM patients [18]. While these conditions may co-exist, some interact, through either shared risk factors or pathophysiology; or one disease influencing susceptibility and outcomes of the other. For example, DM is associated with a 2–3 fold higher risk of TB [19]. Further research is therefore required to evaluate the proportion of TB cases attributable to DM in this high HIV/TB/DM setting.

MM was lower overall in HIV/ART patients compared to patients not on ART with unknown HIV status. However when stratified by age, we noted that in the younger age groups (18–35 and 36–45 years), MM was higher in HIV/ART patients, in particular, there was a higher prevalence of HPT, DM and TB. One possible reason for this difference is the previously reported association between HIV/ART and premature and accelerated ageing [20]. This could also be due to increased awareness of NCD among HIV/ART patients, and possibly increased access to NCD screening in ART clinics. Obesity in HIV-infected patients is an emerging issue in South Africa; with some antiretroviral drugs, such as non-nucleoside reverse transcriptase inhibitors currently in use in South Africa, contributing to lipodystrophy and truncal obesity, increasing the risk of DM, HPT, and metabolic syndrome [10, 21]. A study of MM in HIV-infected patients in the United States found a prevalence of MM of 65%, with prevalence increasing with increasing body mass index (BMI) [22]. In the 46–55 age group, while HPT prevalence was similar between groups, DM prevalence was higher in the HIV/ART group; possibly highlighting a previously reported association between an increased risk of dysglycaemia in HIV-infected patients on ART [21].

Multimorbidity results in complex disease patterns that may have multiplicative, and not merely additive, consequences on health outcomes; and could diminish patients’ ability to manage their condition and enact behavior changes that may be required to improve health. This increasing complexity impacts on both health services, through more intensive health care requirements, and on health providers, with an increased requirement for integrated generalist care at the primary care level. This changing pattern of disease will therefore require health policy and interventions that differ from traditional vertical approaches and single disease management such as integrated management of chronic disease patients considered to be stable [23]. Although there is data paucity on the cost benefit of integrated systems, existing data suggests that integrating chronic disease services into existing HIV care may improve cost effectiveness [24]. For example, established systems for the delivery of ART and TB medications could be adapted to include essential drugs required for NCD management in order to streamline healthcare delivery and potentially improve adherence to chronic medications [25]. Similarly, funding mechanisms that usually fund NCD research could facilitate further research into integrated management building on existing HIV care infrastructure established through HIV funding mechanisms such as the President’s Emergency Plan for AIDS Relief (PEPFAR) [26].

Furthermore the associative patterns of MM described in this study suggest active bidirectional and targeted screening for these conditions should be implemented. Routine active screening is likely to result in an even higher burden of diagnosed co-morbidities in the short term, but diagnosis and intervention at an earlier stage may ultimately result in reduced overall cost. The impact of active screening on the health care system should therefore be evaluated. Beyond these direct interactions, MM and the associated increased complexity could also influence the psychological state or patients’ beliefs and values, influencing decision-making and acceptability of treatment options, and adherence to treatment [27].

### Strengths and limitations

A significant strength of this study is the use of the unique patient identifier number which enabled any treatment prescribed within the public health system in the Western Cape province, even if outside the primary care clinic, to be identified and included in the study. The availability of linked records in a public sector primary care setting is rare both within South Africa and sub-Saharan Africa. This study utilized data from routine databases of prescribed drugs. Diagnoses could therefore not be verified. As a result, only patients with diagnosed chronic diseases receiving treatment for the selected diseases were identified and included in this study. This could underestimate the prevalence of the individual conditions and MM. Patients in chronic care for ART, HPT and DM are plausibly more likely than TB patients to have blood pressure and urine glucose measured over time due to regular clinic visits where routine observations include these measurements; while HIV testing is routinely performed in TB but not HPT and DM clinics. Therefore ascertainment bias with the potential for under ascertainment among TB patients could be a factor. Similarly, in the context of high HIV prevalence in this setting, as emphasized in the methodology, it is important to interpret the HIV stratification within the context of ART versus non-ART as the ‘non-ART/HIV unknown’ subset are likely to include HIV-infected patients who have not been diagnosed or who are not on ART. Another potential limitation was the use of prescribed drugs as a proxy for diagnosis. Whilst medications for DM, HIV, and TB are relatively specific to these diseases, the prescription of hydrochlorthiazide, enalapril or amlodipine may be prescribed for cardiovascular diseases and may not be specific to HPT. However, given the high prevalence of HPT of all chronic diagnoses in primary care [14], and the prescription patterns of doctors at the primary care level, we are confident that this proxy is a valid estimate of HPT. Prior to this study, to confirm prescription patterns in a primary care setting, we conducted a folder review of 100 patients attending another primary care clinic in Khayelitsha and found that these 3 drugs accounted for all HPT patients reviewed.

From a health system perspective, despite these limitations, this study highlights the significant burden of MM among patients receiving chronic disease care at the primary health care level.

## Conclusions

We demonstrated a high prevalence of chronic infectious and non-communicable diseases confirming the epidemiological transition in this peri-urban informal township in South Africa. This study significantly contributes to knowledge about the complex interdependencies in multimorbid diseases in South Africa, a middle-income country. The patterns of MM shown suggest that current models of health care delivery need to be re-examined and patient-centred models of integration evaluated including bidirectional screening of commonly co-morbid conditions in routine clinical practice. Furthermore, research into possible causal underlying mechanisms where unknown; and the implications for diagnosis and treatment, adherence, health outcomes, and capacity for behavior change is required.

## Abbreviations

ID: Infectious Diseases
NCD: Non-communicable diseases
MM: Multiple morbidities
TB: Tuberculosis
DM: Diabetes
HPT: Hypertension
ART: Antiretroviral therapy
LMIC: Low and middle-income countries
CDU: Chronic Disease Dispensing unit
PMI: Patient master index
IQR: Interquartile range
CI: Confidence interval
